# Comprehensive effective and efficient global public health surveillance

**DOI:** 10.1186/1471-2458-10-S1-S3

**Published:** 2010-12-03

**Authors:** Scott JN McNabb

**Affiliations:** 1 Emory University, Rollins School of Public Health, Atlanta, GA, USA

## Abstract

At a crossroads, global public health surveillance exists in a fragmented state. Slow to detect, register, confirm, and analyze cases of public health significance, provide feedback, and communicate timely and useful information to stakeholders, global surveillance is neither maximally effective nor optimally efficient. Stakeholders lack a globa surveillance consensus policy and strategy; officials face inadequate training and scarce resources.

Three movements now set the stage for transformation of surveillance: 1) adoption by Member States of the World Health Organization (WHO) of the revised International Health Regulations (IHR[2005]); 2) maturation of information sciences and the penetration of information technologies to distal parts of the globe; and 3) consensus that the security and public health communities have overlapping interests and a mutual benefit in supporting public health functions. For these to enhance surveillance competencies, eight prerequisites should be in place: politics, policies, priorities, perspectives, procedures, practices, preparation, and payers.

To achieve comprehensive, global surveillance, disparities in technical, logistic, governance, and financial capacities must be addressed. Challenges to closing these gaps include the lack of trust and transparency; perceived benefit at various levels; global governance to address data power and control; and specified financial support from globa partners.

We propose an end-state perspective for comprehensive, effective and efficient global, multiple-hazard public health surveillance and describe a way forward to achieve it. This end-state is *universal, global access to interoperable public health information when it’s needed, where it’s needed.* This vision mitigates the tension between two fundamental human rights: first, the right to privacy, confidentiality, and security of personal health information combined with the right of sovereign, national entities to the ownership and stewardship of public health information; and second, the right of individuals to access real-time public health information that might impact their lives.

The vision can be accomplished through an interoperable, global public health grid. Adopting guiding principles, the global community should circumscribe the overlapping interest, shared vision, and mutual benefit between the security and public health communities and define the boundaries. A global forum needs to be established to guide the consensus governance required for public health information sharing in the 21^st^ century.

## Current state

At a crossroads and fragmented, global public health surveillance is defined as the ongoing systematic collection, analysis, and interpretation of health data, essential to the planning, implementation, and evaluation of public health practice, closely integrated to the dissemination of these data to those who need to know and linked to prevention and control [1,2]. Global, siloed surveillance systems are often slow to detect, register, confirm, analyze, and report cases of public health significance, provide feedback, and communicate timely and useful information to stakeholders [3]. Further, they are neither maximally effective nor efficient in guiding (and being guided by) appropriate interventions. As an essential public health function, surveillance is relatively poorly supported in terms of a global consensus policy, strategy, and governance model and adequate training and resources [4].

While the promise and intuitive added value of integrating public health surveillance highlighted in earlier reports [5] still remains valid (e.g., enhanced timeliness, increased completeness), many more resources and focused effort is required to fulfill this dream that eludes even some of the more advanced and wealthy countries.

New movements have now come to the fore to expedite tomorrow’s digital, paperless public health surveillance workplace and promote comprehensive surveillance. There is strategic merit in conceiving a vision of the end state for comprehensive, global, multiple-hazard public health surveillance; one that acknowledges the challenges and identifies steps to overcome them. We propose here a perspective for comprehensive, effective and efficient global, multiple-hazard public health surveillance and describe a way forward to achieve it.

## Global movements

Three important movements now set the stage to achieve comprehensive, effective and efficient global, multiple-hazard public health surveillance:

1. The adoption of the revised International Health Regulations [IHR(2005)] by all World Health Organization (WHO) Member States, which includes national obligations to achieve a set of core surveillance and response capacities to prevent the international spread of disease [6];

2. The maturation of information sciences (e.g., public health informatics [PHI]) capabilities and the remarkable penetration of information technologies (IT) to the most distant parts of the globe [7]; and

3. A consensus that both the security and public health communities have overlapping interests and mutual benefits to collaborate in supporting the development of essential public health functions, especially public health surveillance [8].

These movements offer the practical opportunity to empower, enhance, and enjoin global public health surveillance, as never before.

### Movement 1 – Adoption of IHR(2005)

The IHR(2005) constitutes the WHO’s legal and operational framework for activities around prevention and control of the international spread of disease, regardless of origin or intent (e.g., chemical and radio-nuclear sources, as well as biological). The adoption of IHR(2005) by WHO Member States challenges them in a new and urgent way to assess and strengthen core surveillance capacities [9]. This agreement also provides the policy context to uniformly assess these capacities [10].

### Movement 2 – The rise of public health informatics (PHI)

Information sciences and IT both used in a public health setting (i.e., public health informatics) are a means to an end; the end being the achievement of effective and efficient public health surveillance. Concerned with effective and efficient collection, collation, transmission, analyses, visualization, storage, and retrieval of electronic data, the scientific discipline of PHI has emerged to leverage the inherent merits found in IT and computer science technology. But this merger yields more than the sum of its individual parts—it has the potential to enhance the transformation to a 21^st^ century global public health surveillance digital (paperless) workplace. By identifying and defining standards and making them easier to apply, PHI adds value to efforts already performed by public health practitioners at all health levels. This added value derives from the inherent ability of PHI to facilitate digital communication in a more robust, efficient, and standards-based manner.

Over the past ten years, many public health processes have been improved by PHI solutions [11]. These improved processes include the increased quantity and timeliness of mandatory case reporting; decreased data-entry burden on public health programs; provision of tools needed for emergency preparedness (e.g., rapid awareness of new cases, linkages to automatic alerting systems for public health personnel); and management support necessary during outbreak situations [12].

PHI also offers paper-to-digital conversion techniques and tools that empower and enable epidemiologists and surveillance practitioners to work better, faster, and cheaper [13] by providing health information, including any information about individuals demonstrated to be related to health (e.g., medical records, laboratory reports, behavioral risk factors, medical examiner and vital records, school records) that is more complete, specific, and timely [14-16].

### Movement 3 – Alignment of security and public health

The third movement aligns the overlapping, mutual interests of the security and public health communities around the domain of public health surveillance. It forces policy discussion, increases focus, as well as provides sources to drive progress. The WHO’s 2007 World Health Report, “A safer future: global public health in the 21^st^ century”, addresses the interface of health and security [17]. The following points are noted in the foreword: “Given today’s universal vulnerability to [internationally significant health and security] threats, better security calls for global solidarity… as the determinant and consequences of health emergencies have become broader, so has the range of players with a stake in the security agenda … successful implementation of the IHR(2005) serves the interests of politicians and business leaders as well as the health, trade and tourism sectors.”

Additionally, World Health Assembly Resolutions 54.14 and 55.16, respectively, requested the WHO to “provide technical support to Member States for developing intervention programmes that prevent epidemics and respond to communicable disease threats and emergencies, particularly with regard to epidemiologic investigations, laboratory diagnoses and community and clinical management of cases” and “to continue, in consultation with relevant intergovernmental agencies and other inter national organizations, to strengthen global surveillance of infectious diseases, water quality, and food safety, and related activities such as the revision of the International Health Regulations and development of WHO’s food safety strategy, by coordinating information gathering on potential health risks and disease outbreaks, data verification, analysis and dissemination, by providing support to laboratory networks, and by making a strong contribution to any international humanitarian response, as required.” [18,19].

The WHO plays a role in the international response to accidental or deliberate use of biological and chemical agents or radio-nuclear materials that affect health. They have a vision for international public health security, ready to respond collectively to the threat of epidemics and other public health emergencies, both natural and man-made. Correspondingly, WHO has adopted mechanisms for supporting countries and strengthening the international response [10].

Among global public health stakeholders there now seems to exist both the political will and an acknowledged, if yet undefined, overlapping interest and mutual benefit to achieving comprehensive, effective and efficient global, multiple-hazard public health surveillance. There are areas where security and public health interests are seen to be at odds. However, some countries may not wish to share public health information with other countries that may be used for the security or economic advantage of the other nation. These perceptions have been a challenge to global public health surveillance in recent years and may be considered a key challenge going forward.

### Eight keys to comprehensive health protection

**Figure 1:**
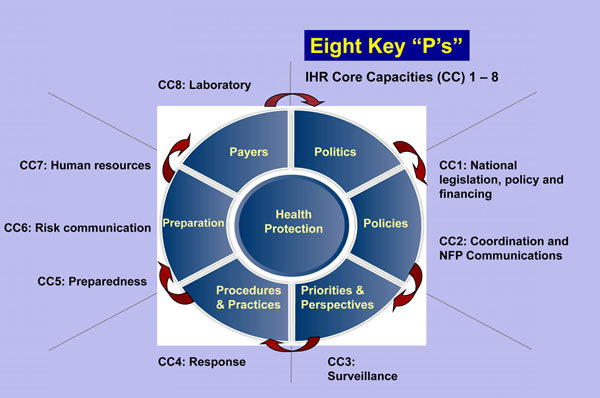
Eight Keys to Comprehensive Health Protection and their Relationship to the Eight Core Capacities of the International Health Regulations 2005.

**Figure 2:**
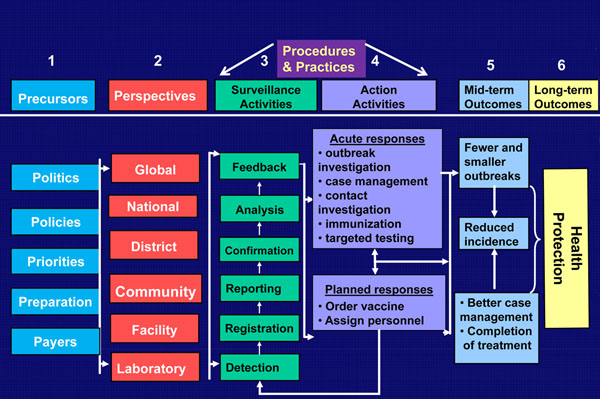
Relationships and Dependencies among the Eight Keys of Comprehensive Health Protection to Comprehensive Effective and Effcient Global Public Health Surveillance.

In order for these three movements to empower and enhance surveillance competencies and lead to the end-state perspective described here, eight prerequisites or conditions should be in place (Figure 1). They include politics, policies, priorities, perspectives, procedures, practices, preparation, and payers (Table 1). While these eight prerequisites have a loosely sequential nature, there are relationships and interdependencies among them that should be acknowledged because of critical linkages to core competencies of public health surveillance and action (Figure 2).

**Table 1:** Eight Keys to Comprehensive Health Protection and their Defining Features.

Key	Defining Features
Politics	public demand; general consensus; mutual interest
Policies	governance; stewardship; respect for human rights and data ownership; best practices
Priorities	shared vision; acceptance of accountability; embracing strategic and system’s thinking; seeing a common way forward; finding synergy; leveraging existing strengths
Perspectives	facility; community; district; regional; national; global
Procedures	review global best practices; assess standard operating procedures; delineate lines of authority; establish channels of communication; intervene and re-evalaute
Practices	assess existing workflow; evaluate effectiveness and effciency
Preparation	training; transparency; forthrightness; one size does not fit all
Payers	local and national governments; global programs

Many challenges (gaps and impediments) exist between the current and end-state described here. They can be disaggregated into technical, logistic, governance, and financial domains. Within the technical domain, single (silo) categorical, disease-specific surveillance systems create the situation where public health practitioners cannot determine relationships between health conditions or co-morbidities (e.g., through data linkages). For example, new tuberculosis (TB) cases could be missed because HIV/AIDS data are not cross-matched with the TB registry. In this situation, surveillance becomes ineffective, because it is incomplete; events that represent a public health threat or that could inform about a potential public health threat are missed. Many times disease-specifi c silos are created and sustained by single funding streams and corresponding program obligations and priorities. They are often then maintained by program corporate cultures. Secondly, appropriate public health information is often not collected because what needs to be measured is often not known [20,21]. In these circumstances, surveillance systems are not flexible enough to respond to new or unusual presentations of disease, events, or conditions (i.e., not easily adapted to changing information needs). Thirdly, current surveillance systems are often not timely (i.e., by the time an event of interest or concern is detected, the opportunity to intervene has passed) [22]. Additionally, some new surveillance systems are neither effective, nor efficient (i.e, the delivery of needed information is dependent on an inordinate number of resources) [23]. The logistic gaps include technical disparities among Member States, such as the cost for internet access and IT infrastructure. Gaps also exist in surveillance practices (e.g., lacking legal or other administrative requirements for mandatory reporting).

There is a critical gap in global governance, under which all Member States would agree to function. Countries now collect and communicate public health information within and outside their natural border. The amount and type of information and willingness to collaborate varies from region to region. While there is a justifiable need to share important public health information that might impact neighboring states (and the IHR[2005] provides the legal and technical framework for public health emergencies of international concern), there still exists some uncertainty about what types of public health information are appropriate, how they should be communicated, and how quickly they should be shared.

Challenges (or impediments) to bridge the gaps include both the lack of trust and perceived benefit at various levels, the lack of a global governance model to address power and control of public health information, and the lack of focused financial support from global partners.

## Conclusions

### End-state perspective for global public health surveillance

Enhancing global public health surveillance in the 21^st^ century involves empowering and enabling existing public health surveillance systems to interoperate (i.e., one information system to communicate with another syntactically – meaning two or more systems are capable of communicating and exchanging data by using specified data formats and communication protocols; and semantically – meaning the ability of computer systems to communicate information and have that information properly interpreted by the receiving system in the same sense as intended by the transmitting system). Surveillance systems should also be enjoined (or integrated – meaning streamlining data collection among systems to reduce redundancy of the data collected where it makes sense to do so). These activities will lead to *universal global access to interoperable public health information when it’s needed, where it’s needed*. In the process sense, interoperable and integrated public health information means achieving effective and efficient public health business practices and workflow empowered and enabled to be better, faster, and cheaper by IT.

Operationally, comprehensive, global public health surveillance means one sign-on access to authorized and necessary public health information. Public health information includes other information – when combined with health-related information – that provides a picture of population or community health. Consumers should have one-stop shopping for public health information; and there should be one source for integration of public health information for all users. This also means one common set of standards for “bringing together” or interoperating existing or new data streams. Most importantly, one size does not fit all.

Demographic, clinical, laboratory and other information about patients with diseases of public health significance should only have to be entered once, saving time and resources. The local, district, national, or inter national health authorities should be able to access real-time health outcome data and perform analyses or take timely and appropriate public health action based on that information. It should be stressed that keeping electronic health information private, confidential, and secure while automatically and immediately electronically communicating public health information to local public health authorities to satisfy mandatory public health reporting purposes is critical. Different reporting systems may be in existence depending on the types of data and information being reported, purpose and urgency of relaying the information, and where the data/information is being reported.

This perspective embraces and mitigates the tension between two fundamental human rights. The first is the human right to privacy, confidentiality, and security of personal health information. This includes any informa-tion about individuals demonstrated to be related to health (e.g., medical records, laboratory reports, behavioral risk factors, medical examiner and vital records, school records) and the right of sovereign, national entities to the ownership and authority over their citizens’ public health information. The second is the human right of individuals to have real-time access to public health information that might impact their lives.

Data management requires much more than investment in technology; it involves how data are created, stored, moved, used, and retired. As opposed to the 20^th^ century replicated database model, a federated model of public health information sharing allows multiple participants to share data without having to give up ownership, thus accommodating universal access to public health information [24]. A federated model is a type of meta-database management system that transparently integrates multiple autonomous database systems into a single one. The constituent databases are interconnected via a computer network, and may be geographically decentralized. Since the constituent database systems remain autonomous, a federated database (or virtual database) is the fully-integrated, logical composite of all constituent databases.

**Figure 3:**
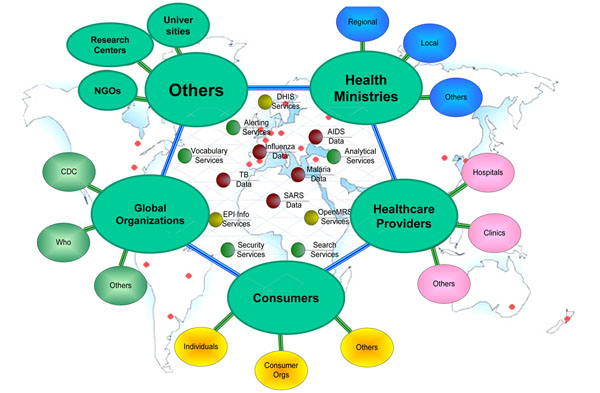
Envisioned Architecture of Global Public Health Grid.

One mechanism to achieve this federated model is through a global public health grid (http://cdc.confex.com/cdc/phin2009/webprogram/Paper21091.html) (Figure 3). The goal of the grid is to improve population health by facilitating timely and reliable global public health information exchange. The global grid has the core principles of long-term sustainability; low barrier to entry (technically, financially and socially); and uses a standards-based approach that is reusable, collaborative, and open source.

## Recommendations

The way forward that addresses the gaps and challenges to achieve the end-state paradigm described here for comprehensive, effective and efficient, multiple-hazard global public health surveillance lies through incorpora-tion of these key principles:

1. People are key and listening is important – true partnership takes this into account.

2. Transparency builds trust and is crucial to success.

3. Mutual respect and mutual benefit are vital and necessary. This includes a full recognition of data owner ship, national sovereignty, and the rights of individual patients.

4. Competence, relevance, and a common language in public health practice are required.

5. A culture of responsible stewardship and quality data is mandatory.

6. While one-size-does-not-fit-all, a set of core capacities does exist [25]. Each Member State should proactively assess its own public health surveillance performance to identify and address gaps.

Adopting these guiding principles, the global community should carefully circumscribe the overlapping interest, shared vision, and mutual benefit of the security and public health communities within the domain of public health surveillance and define the boundaries of those mutual interests. Finally, a global forum should be established to guide consensus governance required for public health information sharing in the 21^st^ century. Once the impediments of power and control of data are recognized, respected, and addressed, universal access to public health information can occur.

## Abbreviations

IHR: International Health Regulations; IT: Information technology; PHI: Public Health Informatics; TB: Tuberculosis; WHO: World Health Organization.

## Competing interests

We confirm that we do not have any conflicts of interest. Drs. McNabb and Chungong have no financial or personal relationships that inappropriately influence (bias) their actions (such relationships are also known as dual commitments, competing interests, or competing loyalties). Neither of us have financial relationships (such as employment, consultancies, stock ownership, honoraria, and paid expert testimony) likely to undermine our credibility. We have no conflicts for other reasons, such as personal relationships, academic competition, and intellectual passion.

As a guest editor, Dr. McNabb will avoid selecting external peer reviewers with obvious potential conflicts of interest—for example, those who work in the same department or institution as any of the authors. He has no personal, professional, or financial involvement in any of the issues he might judge.

## Authors’ contributions

We both contributed to the conceptualization, writing, and editing of this manuscript. We will both defend its findings.
